# Plasma miR-126 Is a Potential Biomarker for Early Prediction of Type 2 Diabetes Mellitus in Susceptible Individuals

**DOI:** 10.1155/2013/761617

**Published:** 2013-12-25

**Authors:** Tao Zhang, Chunfang Lv, Liling Li, Sihan Chen, Shenglin Liu, Changyi Wang, Bing Su

**Affiliations:** ^1^Department of Laboratory Medicine, Shenzhen Nanshan Center for Chronic Disease Control, Shenzhen, Guangdong 518054, China; ^2^Department of Control and Prevention for Chronic Non-Communicable Diseases, Shenzhen Nanshan Center for Chronic Disease Control, Shenzhen, Guangdong 518054, China; ^3^Biomedical Research Institute, Shenzhen PKU-HKUST Medical Center, Shenzhen, Guangdong 518036, China; ^4^Shenzhen Key Laboratory for Translational Medicine of Dermatology, Shenzhen PKU-HKUST Medical Center, Shenzhen, Guangdong 518036, China; ^5^Shenzhen Key Discipline of Dermatology, Peking University Shenzhen Hospital, Shenzhen, Guangdong 518036, China; ^6^Department of Social and Preventive Medicine, School of Public Health and Health Professions, The State University of New York, Buffalo, NY 14214, USA; ^7^Department of Cancer Genetics, Roswell Park Cancer Institute, Buffalo, NY 14263, USA

## Abstract

Type 2 diabetes mellitus (T2DM) is a major public health problem in China. Diagnostic markers are urgently needed to identify individuals at risk of developing T2DM and encourage them to adapt to a healthier life style. Circulating miRNAs present important sources of noninvasive biomarkers of various diseases. Recently, a novel plasma microRNA signature was identified in T2DM. Here, we evaluated the T2DM-related miRNA signature in plasma of three study groups: normal (fasting glucose (FG), 4.8–5.2 mmol/L), T2DM-susceptible (FG, 6.1–6.9 mmol/L), and T2DM individuals (FG, ≥7.0 mmol/L) and tested the feasibility of using circulating miRNAs to identify individuals at risk of developing T2DM. Among the 5 miRNAs included in the signature, miR-29b and miR-28-3p are not detectable. miR-15a and miR-223 have comparable expression levels among three groups. Notably, miR-126 is the only miRNA that showed significantly reduced expression in susceptible individuals and T2DM patients compared to normal individuals, suggesting that miR-126 in circulation may serve as a potential biomarker for early identification of susceptible individuals to T2DM.

## 1. Introduction

Diabetes mellitus (DM) affects more than 350 million people currently and additional 552 million people within the next two decades worldwide [1]. In China, the prevalence of DM has increased markedly in recent years and is now reaching epidemic proportions [2]. The Journal of American Medical Association (JAMA) reported that the number of adult diabetic patients in China has reached 113.9 million, accounting for almost one third of DM patients worldwide, indicating that DM has become a major public health issue in China [3, 4]. The majority of the cases are attributable to T2DM in China, most likely due to major diet and life style changes caused by rapid economic growth. These changes include increased nutrition and reduced physical activities which promote obesity. Along these lines, our recent survey showed that T2DM occurrence in Shenzhen, Guangzhou province, increases rapidly from 4.7% to 6.2% in the past 5 years and reaches an alert level (unpublished data). Overall, DM is becoming a major risk factor for morbidity and mortality worldwide [5]. To date, the causes of DM remain unclear and a definitive cure is still not available [6]. However, the onset and progression of DM will be drastically delayed if preventive interventions could be implemented for highly susceptible T2DM individuals before and during the initial phase of the disease. The development of biomarkers for early detection of DM will help identify individuals at risk of developing DM and greatly improve the care for these individuals and reduce or delay the occurrence and severity of the disease. Hence, the discovery of new biomarkers would enable clinicians to tailor preventive and therapeutic approaches and minimize the expected negative impacts of the disease.

Noncoding RNAs (ncRNAs) are divided into two main categories based on transcript size: small ncRNAs and long noncoding RNAs (lncRNAs) [7, 8]. They are emerging as important new regulators in cancer and other diseases [7, 8]. Small ncRNAs include a broad range of known and newly discovered RNA species, many of which associate with 5′ or 3′ regions of genes. microRNAs (miRNAs) belong to this category. Altered expression of miRNAs has been documented in human diseases [9, 10], prompting an increasing interest in their use as biomarkers for diagnosis and prognosis as well as potential therapeutic targets [11–13]. To date, miRNAs have been widely reported in blood as potential biomarkers for the detection of cancers [10, 14], cardiovascular diseases [12, 15], and kidney diseases [16].

Aberrant expression of miRNAs in DM has been reported recently [17, 18], suggesting a potential for their use as biomarkers for disease diagnosis. In this study, we evaluated the plasma expression patterns of five T2DM-related miRNAs in three study groups: normal individuals (fast glucose (FG), 4.8–5.2 mmol/L), T2DM susceptible individuals (FG, 6.1–6.9 mmol/L) and diagnosed T2DM patients (FG, ≥7.0 mmol/L), and tested the feasibility of clinical application of circulating miRNAs in early prediction of T2DM in susceptible individuals.

## 2. Materials and Methods

### 2.1. Participants

In this cohort, 90 Han Chinese individuals, aged 42–75 years, receiving routine physical exams were recruited by the Outpatient Department of Laboratory Medicine, Chronic Disease Hospital of Nanshan District in Shenzhen, China. They were recruited to 3 study groups (30 subjects/group): normal individuals (fasting glucose (FG), 4.8–5.2 mmol/L), T2DM susceptible individuals (FG, 6.1–6.9 mmol/L), and diagnosed T2DM patients (FG, ≥7.0 mmol/L). Individuals in the normal and susceptible groups with the following conditions were excluded from the study: common diabetic complications such as retinopathy, nephropathy and cardiovascular disorders which may pose latent effect on miRNA expression. Individuals who had previously been diagnosed with DM or had any history of medication for 6 months prior to the study were also excluded. T2DM was diagnosed based on the combination of several parameters: FG level higher than 7.0 mmol/L and 2 h plasma glucose (PG) higher than 11.1 mmol/L in oral glucose tolerance test (OGTT), or diagnosed by other hospitals. Written consents were obtained from all subjects prior to the recruitment and the study protocol was approved by the Ethics Committee of Chronic Disease Hospital of Nanshan District. The clinical characteristics of the subjects are listed in Table 1.

### 2.2. RNA Isolation and Real-Time Quantitative RT-PCR (qRT-PCR)

Peripheral blood was collected via venipuncture into tubes containing sodium EDTA, centrifuged at 1000 g for 5 min, and the plasma was carefully transferred into RNase-free tubes and stored at −80°C until use. Total RNA containing miRNAs was isolated from plasma using isothiocyanate-phenol/chloroform extraction procedures. cDNA synthesis was performed with RevertAid First Strand cDNA Synthesis kit according to the manufacturer's instructions (Thermo). The SYBR Premix DimerEraser kit (TaKaRa, Shiga, Japan) was used in real-time PCR for relative quantification of miRNAs with miR-238 as an internal control. qRT-PCR was performed on a Bio-Rad CFX-96 real-time PCR system (Bio-Rad, Hercules, CA). The sequences of the PCR primers used were listed in Table 2.

### 2.3. Statistical Analysis and Algorithm of Figure Generation

The differences in the expression of plasma miRNAs among the groups were analyzed with Student's test concerning clinical parameters as median age and sex. All statistical analyses were done with STATA 10.0 (StataCorp LP, College Station, TX). *P* < 0.05 was considered statistically significant. miRNA expression heat-map in Figure 2(a) was generated by matrix2png (http://chibi.ubc.ca/matrix2png/bin/matrix2png.cgi).

## 3. Results

### 3.1. Clinical Characteristics of Study Subjects

When recruited, the average ages of normal individuals, T2DM susceptible individuals, and diagnosed T2DM patients were 61 ± 9, 62 ± 8, and 63 ± 8.6 years, respectively. No significant difference in age and sex distribution was found among three study groups (*P* > 0.05) (Table 1).

### 3.2. Circulating miRNA Expression in Plasma Samples

First, we validated the feasibility of miRNA detection in plasma samples of three study groups. Total RNA containing miRNAs was isolated from plasma samples and quantified for the subsequent qRT-PCR analysis. No significant difference in RNA concentrations was discovered among the groups (data not shown).

**Figure 1:**
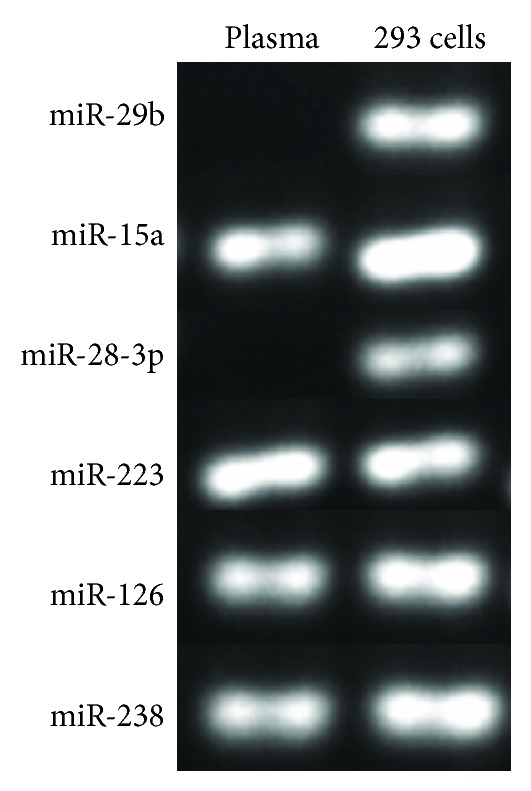
Detection of miRNAs in plasma samples of normal, T2DM-susceptible individuals, and diagnosed T2DM patients as well as 293 cells. RNA was extracted from plasma samples of the 3 groups and 293 cells. miRNAs were assessed by qRT-PCR assays. The samples with an average qRT-PCR result of Ct < 40 were considered as positive. The qRT-PCR products were visualized by 2% agarose gel electrophoresis.

Up to date, only one study examined miRNA expression in plasma samples of T2DM patients [17]. From the miRNA signature identified in the study, we selected five DM-associated miRNAs (miR-29b, miR-15a, miR-28-3p, miR-223, and miR-126) [17] to evaluate their expression in plasma samples of normal, susceptible individuals, and diabetic patients. miR-238 is used as an internal control. The samples with a qRT-PCR result of Ct < 40 were considered as positive. We found out that 3 out of 5 selected miRNAs were detectable in plasma from all three groups (Figure 1). However, inconsistent with the reported finding [17], miR-29b and miR-28-3p were undetectable in any group (Figure 1). To validate the miRNA qRT-PCR assays, we examined the expression of these miRNAs in 293 cells. All of the 5 miRNAs were readily detected in 293 cells, which demonstrated the reliability of the procedure and primers (Figure 1). Therefore, the failure to amplifying miR-29b and miR-28-3p could simply indicate their absence or very low expression levels in the plasma samples collected.

### 3.3. miR-126 Expression Was Significantly Downregulated in Plasma Samples of T2DM Susceptible Subjects and T2DM Patients

**Figure 2:**
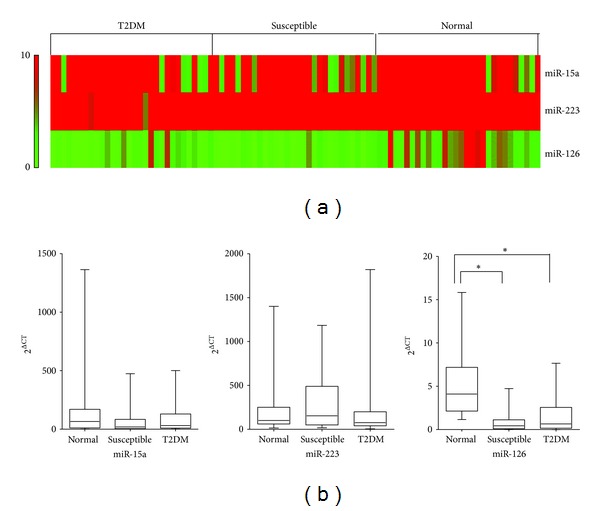
Circulating levels of miR-15a, miR-223, and miR-126 in plasma samples of normal, T2DM-susceptible individuals, and diagnosed T2DM patients. qRT-PCR was performed with RNA extracted from plasma samples from 3 groups. (a) The heat-map depicts the expression of the 3 miRNAs in the 90 plasma samples. (b) The graph showed that relative plasma miRNAs.

Next, we compared the expression of detectable miRNAs in three groups. No statistically significant difference was found in the expression of miR-15a and miR-223 among the groups. In contrast, the expression level of miR-126 was significantly lower in plasma samples of the susceptible and T2DM groups compared with the normal group (*P* < 0.01) (Figures 2(a) and 2(b)). No significant difference was found between miR-126 levels in plasma samples of the susceptible and T2DM groups (Figures 2(a) and 2(b)).

We further compared the expression of miR-126 in different sex and age groups. No statistical difference in miR-126 expression was found between male and female participants (*P* = 0.517). Dividing the subjects into two age groups by the median age (65), there was no statistical difference in miR-126 expression between these two age groups either (*P* = 0.526).

### 3.4. The Association of Circulating miR-126 with FG Concentration

To evaluate whether miR-126 expression associates with blood glucose levels, one way-ANOVA test was conducted. The result indicated that different FG levels associate with distinct miR-126 expression levels (*P* < 0.0001). Further, Bonferroni's multiple comparison test showed that miR-126 expression was significantly different between the subjects with low and medium FG levels (4.8–5.2 versus 6.1–6.9 mmol/L) (*P* < 0.05, 95% CI of diff = 2.82–6.04). miR-126 expression also differed between the patients with low and high FG levels (≥7 mmol/L) (*P* < 0.05, 95% CI of diff = 1.99–5.21). However, no difference in miR-126 expression was found between patients with medium and high FG levels (*P* > 0.05, 95% CI of diff = −2.44–0.780). Together, these results support an association between fasting glucose levels and miR-126 expression in circulation.

## 4. Discussion

DM affects more than 350 million people worldwide in 2011 [1]. The prevalence of DM in Asia has increased significantly in recent years with a disproportionate burden in young and middle-aged population [19]. The situation is considered especially urgent in China because it is becoming a major public health problem. Despite the widely publicized report of 9.7% prevalence for diabetes and 15.5% for prediabetes in the 2007 survey [2], the latest epidemiologic study suggests that the estimated prevalence has increased to 11.6% for diabetes and 50.1% for prediabetes [3]. Unfortunately, the epidemic of diabetes and prediabetes in China has shown no sign of abating [3].

Due to complex etiology which involves genetics, diet, life style, and environmental factors, DM is posing diagnostic and therapeutic challenges. The existing traditional biochemical markers for DM including levels of glucose, triacylglycerol, cholesterol, lipoproteins, and HbA1c may predict the development of DM a few years prior to disease manifestation. However, these biomarkers are not specific for DM and cannot be used to assess disease susceptibility in the general population. Hence, the discovery of new biomarkers that could be used to identify individuals at risk and thereby provide early intervention to delay the development and progression of DM is urgently needed.

miRNAs belong to a class of small noncoding RNAs that function as translational repressors by partially pairing to the 3′ untranslated region of target mRNAs. The idea of using circulating miRNAs as biomarkers is fairly new and was first proposed for detecting various types of cancer [20]. Circulating miRNAs are very stable and resistant to ribonucleases, freezing/thawing cycles, and other drastic experimental conditions [21]. Consequently, serum or plasma samples can be stored at −20°C or −80°C for several months without notable degradation of miRNAs, which supports the utility of miRNAs as ideal candidate biomarkers [22].

The miRNA expression profiles in serum samples of pre-diabetic Han Chinese individuals or T2DM patients have recently been analyzed [23]. The results showed increased expression of 7 diabetes-related miRNAs (miR9, miR29a, miR30d, miR34a, miR124a, miR146a, and miR375) in T2DM patients compared with pre-diabetic or T2DM susceptible individuals [23]. However, no difference in miRNA expression was observed between individuals with normal glucose tolerance and pre-diabetic individuals, indicating that these serum miRNAs cannot be used for the prediction of susceptibility to T2DM. Zampetaki and colleagues systematically examined the plasma miRNA expression profiles in patients with T2DM by microarray screening and revealed a unique plasma miRNA signature for DM [17]. This pioneer study demonstrates the potential of plasma miRNAs as diagnostic and prognostic biomarkers for T2DM.

In this study, we focused on the expression patterns of 5 diabetes-related signature miRNAs identified by Zampetaki's group and investigated the potential of using plasma miRNAs as a noninvasive tool for blood based prediction of highly susceptible individuals to develop T2DM. We present novel findings that plasma miR-126 level is significantly lower in susceptible individuals and established T2DM patients in comparison to normal individuals, and the decreased miR-126 level is inversely associated with the development of DM. Interestingly, miR-126 expression is not associated with the sex and age of patients. Together, our results support miR-126 as a potential independent risk factor and diagnostic biomarker of susceptible individuals to developing T2DM. Compared to the existing traditional biochemical markers, our proposed miR-126 is more specific to DM and may be able to facilitate early identification of DM risk since its expression level is significantly lower even in the DM susceptible individuals. miRNAs are also more stable and less likely to be affected by diet and physical activities before the blood sample collection. Even though DM is affected by multiple complex factors, such as genetics, environment, diet, and life style, undoubtedly, genetic factor contributes the most to its occurrence. Hence, the identification of genetic markers, such as miRNAs, will yield new and reliable approaches to prevent DM through early identification of individuals at risk.

In conclusion, we have shown that miR-126 is significantly reduced in plasma samples of T2DM susceptible individuals and T2DM patients. These findings suggest that detection of circulating miR-126 can be developed into a noninvasive and rapid diagnostic tool for the prediction of susceptible individuals to developing T2DM. Furthermore, screening a large cohort of plasma samples of susceptible subjects will further determine the usefulness of miR-126 expression as a potential pre-DM biomarker.

## Figures and Tables

**Table 1:** Clinical characteristics of the study subjects.

Characteristics	Normal	Susceptible	DM	*P* value
Sex				
Male	16	9	16	
Female	14	21	14	>0.05
Ages (yr)				
Mean	61 ± 9	62 ± 8	63 ± 8.56	
Range	42–75	47–72	42–73	
Median	62	64	65.5	>0.05
FG	4.86 ± 0.36	6.45 ± 0.24	10.8 ± 2.9	<0.01

**Table 2:** Nucleotide sequences of primers for miRNA qPCR.

miRNA	Primer	Sequence
miRNA15a	RT	GTCGTATCCAGTGCGTGTCGTGGAGTCGGCAATTGCACTGGATACGAC CACAAAC
	F	GGCTAGCAGCACATAATGG
miRNA29b	RT	GTCGTATCCAGTGCGTGTCGTGGAGTCGGCAATTGCACTGGATACGAC AACACTG
	F	GGCTAGCACCATTTGAAATC
miRNA28-3p	RT	GTCGTATCCAGTGCGTGTCGTGGAGTCGGCAATTGCACTGGATACGAC TCCAGG
	F	GCGCACTAGATTGTGAGCT
miRNA223	RT	GTCGTATCCAGTGCGTGTCGTGGAGTCGGCAATTGCACTGGATACGAC TGGGGT
	F	GGCCTGTCAGTTTGTCAAA
miRNA126	RT	GTCGTATCCAGTGCGTGTCGTGGAGTCGGCAATTGCACTGGATACGACCGCATT
	F	GCGTCGTACCGTGAGTAAT
miR238	RT	GTCGTATCCAGTGCGTGTCGTGGAGTCGGCAATTGCACTGGATACGACTCTGAA
	F	AGCCTTTGTACTCCGATGC
HasmirR	R	GTGCGTGTCGTGGAGTC
